# Color Difference and Memory Recall in Free-Flying Honeybees: Forget the Hard Problem

**DOI:** 10.3390/insects5030629

**Published:** 2014-07-30

**Authors:** Adrian G. Dyer, Jair E. Garcia

**Affiliations:** 1 Department of Physiology, Monash University, Clayton VIC 3800, Australia; 2 School of Media and Communication, RMIT University, Melbourne VIC 3001, Australia; E-Mail: jair.garcia@rmit.edu.au

**Keywords:** differential, absolute, conditioning, flower

## Abstract

Free-flying honeybees acquire color information differently depending upon whether a target color is learnt in isolation (absolute conditioning), or in relation to a perceptually similar color (differential conditioning). Absolute conditioning allows for rapid learning, but color discrimination is coarse. Differential conditioning requires more learning trials, but enables fine discriminations. Currently it is unknown whether differential conditioning to similar colors in honeybees forms a long-term memory, and the stability of memory in a biologically relevant scenario considering similar or saliently different color stimuli. Individual free-flying honeybees (N = 6) were trained to similar color stimuli separated by 0.06 hexagon units for 60 trials and mean accuracy was 81.7% ± 12.2% s.d. Bees retested on subsequent days showed a reduction in the number of correct choices with increasing time from the initial training, and for four of the bees this reduction was significant from chance expectation considering binomially distributed logistic regression models. In contrast, an independent group of 6 bees trained to saliently different colors (>0.14 hexagon units) did not experience any decay in memory retention with increasing time. This suggests that whilst the bees’ visual system can permit fine discriminations, flowers producing saliently different colors are more easily remembered by foraging bees over several days.

## 1. Introduction

Honeybees are effective pollinators of flowering plants, including many species of high economic value [1]. Individual bees visit many flowers to collect small amounts of nutrition in the form of nectar and/or pollen, and in the process of visiting multiple plants incidentally transfer some conspecific pollen to facilitate pollination [2]. To identify the most profitable flowers, and avoid non-rewarding flowers like mimics [3], bees make use of a number of cues including olfaction [4], shape [5], symmetry [6] and color [7,8] information. The high value of color as a cue for bees finding profitable flowers is evidenced by the evolution of many flowering species to produce spectral signals that are best discriminated by the visual system of honeybees [9], or other trichromatic bees that share very similar color discrimination characteristics [10,11]. Indeed, it is known that the evolution of flower colors to fit bee discrimination is a one-sided story, as hymenoptera trichromatic vision is phylogentically ancient and predates the evolution of flower colors [12].

One feature of honeybee foraging behavior that is of high value for facilitating the efficient distribution of pollen is the tendency of individual pollinators to be flower constant and continue visiting the same type of flower whilst it is available [13]. The mechanisms underlying flower constant behavior are not fully understood, but it is likely that by remaining constant to one type of profitable flower enables individual bees to benefit from reduced interference sensitivity of short-term memory [13,14]. Color is an important cue in promoting flower constancy by individual bees, which reinforces the evolutionary pressure on flowering plants to produce spectral signals that are easily identified by pollinators [10,15].

The visual system of honeybees contains three types of spectrally different photoreceptors that are maximally sensitive in the UV (344 nm), blue (436 nm) and green (544 nm) parts of the electromagnetic spectrum [12,16]. The capacity of honeybees to discriminate colors was first demonstrated 100 years ago by Karl von Frisch [17]. Subsequently is has been shown that bees have trichromatic vision [18] with best discrimination at about 400 and 500 nm where photoreceptor sensitivities overlap [19], and bees learn saliently different spectral colors within a few trials [20]. Such learning fits both with regions of the spectrum for which discrimination is best, and flower cues that tend to be more associated with profitable nectar rewards [21]. However, in complex natural conditions the color of flowers from competing plant species may not always be saliently different, thus a significant problem that the visual system of the bee has to deal with is reliably discriminating between similarly colored flowers, especially as mimic flowers try to gain visits by deception without offering rewards [3,22]. To deal with this problem honeybees can learn colors in different ways depending upon the nature of the problem presented to them. If only a single type of rewarding color is presented (termed absolute “conditioning”) bees learn the target color quickly, but cannot make fine discriminations between the target and similar colors; however if a target color is learnt in the presence of similar non-rewarding distractors (termed “differential conditioning”) honeybees slowly learn to make fine color discriminations [23,24].

An important component of flower learning and subsequent flower constancy behavior is the capacity of an individual bee to recall from memory a color for which a positive association has been made. For saliently different spectral colors it has been shown that whilst bees that have had three or more learning trials can very reliably recall the information for over two weeks, bees that only have a single learning trial have a memory trace that fades within a few days [25], showing the existence of different memory phases in honeybees [26]. However, other work suggests that the dynamics of bee memory may also depend on factors like the complexity of a learning task and the salience of cues, including color, which might also contribute to the formation of different memory phases [27]. In the current study we consider how robust the honeybee memory is for a task of correctly choosing between similar colored paper stimuli following differential conditioning, when such information must be recalled several days later in a natural foraging context. Specifically, we hypothesise that following differential conditioning to a perceptually difficult colour task that if honeybees do not establish a long term memory then accuracy of decisions should return to chance expectation within a day. We also consider if memory for either a perceptually easy or difficult task changes with extended time, although in reality there is a continuum of difficulty for bees discriminating between dissimilar or similar color stimuli [15]. The findings present new insights for both the rich plasticity of honeybee memories [27], and how pollinators make decisions using color information in complex environments [28].

## 2. Experimental Section

### 2.1. General Conditions

Experiments were conducted with free-flying honeybees (*Apis mellifera*) at the Jock Marshall Reserve, Monash University, Australia. Honeybees were individually recruited from a gravity feeder that provided 10% (vol.) sucrose solution. At a testing site positioned 10m from the gravity feeder, each bee was trained using a 50 cm rotating screen [5] that vertically presented four stimuli; two color targets (CS+: associated with 30% sucrose) and two color distractors (CS−: associated with tap water) [29]. Each bee was trained on a separate day. There were two experimental groups: Group 1 trained and tested with similar colors, and Group 2 trained and tested with dissimilar colors.

#### 2.1.1. Group 1 Testing with Similar Colors

**Figure 1:**
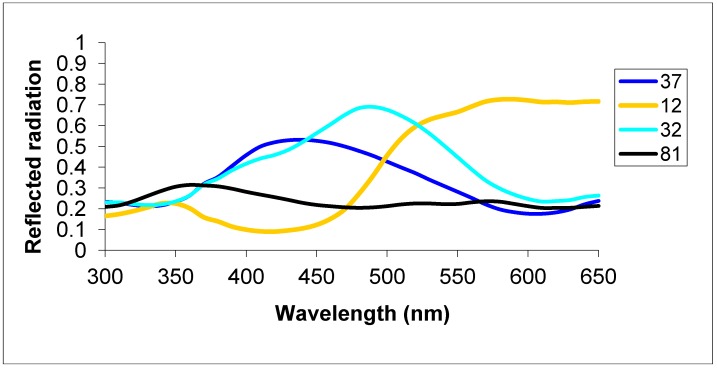
Spectral reflectance of the four Tonpapier (Baehr, Germany: code number shown) cards used as stimuli. The colour of the lines approximately matches the colour of the cards considering human trichromatic vision.

In Group 1 each bee was first provided with absolute conditioning with two color targets (CS+ size = 6 × 6 cm; 130 GSM Tonpapier No. 32, Baehr, Germany: see Figure 1 for spectral reflectance) for 30 trials, whilst two hangers additionally each presented a 6 × 6 cm neutral grey card (No. 81) that matched the background of the rotating screen and allowed for scoring of choice frequency visits to respective hangers. Absolute conditioning implies the visual system learns a target colour independent of other stimuli in a training situation, for example if a Y-maze contains a colour target on a grey background in one arm and the alternative arm only contains a grey background, then only the target colour is learnt [23]. The color distance between these stimuli was determined using a hexagon color model of bee vision [30] and was 0.14 hexagon units, which is a color bees can easily learn [31]. This first stage is essentially absolute conditioning of an easy task to establish bees could learn; indeed all six bees in this group were very accurate at this pre-training task (mean accuracy = 94.8% ± 7.3% s.d.). Following absolute conditioning, each bee then received two non-rewarded tests, each lasting for 30 landings, which were interspersed with a short refresher bout to encourage motivation. The first test presented the training target color and a novel yellow color (No. 12; color distance = 0.23) which confirmed that the bee had learnt the target color (accuracy = 95.8% ± 4.7% s.d.; 1 sample *t*-test, *t* = 23.978, *p* < 0.001); this yellow color was used in only the first test. The second test presented a perceptually similar target color (Tonpapier No. 37; color distance *versus* No. 32 = 0.06 hexagon units) and in this case bee choices for the target color were at chance levels (accuracy = 51.2% ± 6.6% s.d.; 1 sample *t*-test, *t* = 0.460, *p* = 0.665); thus the bees generalized these similar colors following absolute conditioning establishing the perceptual difficulty of this visual task for the main experiment.

Each of the six bees in Group 1 next received differential conditioning [23,24,29] for 60 choices to the perceptually difficult color task (No. 32 *vs.* No. 37). This training took 3–5 h per bee and so only one bee was tested per day. Following the differential conditioning, each bee then received a test with fresh papers of these perceptually similar stimuli, which was defined as accuracy for day 1. On subsequent days a test bee was again recruited to the test apparatus with 30% sucrose, and provided with five landings as absolute conditioning on rewarded target stimuli (No. 32) to insure motivation [27], satiated, and allowed to return to the hive. This enabled individually retesting one marked bee at a time, as all other bees remained at the gravity feeder. When the selected “test bee” returned to the test site it was given a test with fresh perceptually similar stimuli, and this procedure was repeated for as long as test bees were available for up to two weeks. During testing a bee never received any additional differential conditioning with stimuli. We thus classify this as testing memory for the task of colour discrimination as no additional differential conditioning including both CS+ and CS− was provided after day 1. We thus define memory as a capacity to recall a learnt task in a complex environment after a period of not less than 2 h [27].

#### 2.1.2. Group 2 Testing with Dissimilar Colors

Each of the six independent bees in Group 2 was trained and tested with dissimilar colors. The target was Tonpapier No. 32 turquoise; each bee initially received absolute conditioning for 30 trials as described above in Section 2.1.1. Following this training each bee received a test presenting the two target stimuli and two yellow distractors (Tonpapier No. 12) on day 1 of training and on subsequent days to allow for a comparison to the data for Group 2. The color distance between the blue and yellow stimuli was 0.23 hexagon units, which is a very easy color task for bees to discriminate [22,31].

## 3. Results and Discussion

**Figure 2:**
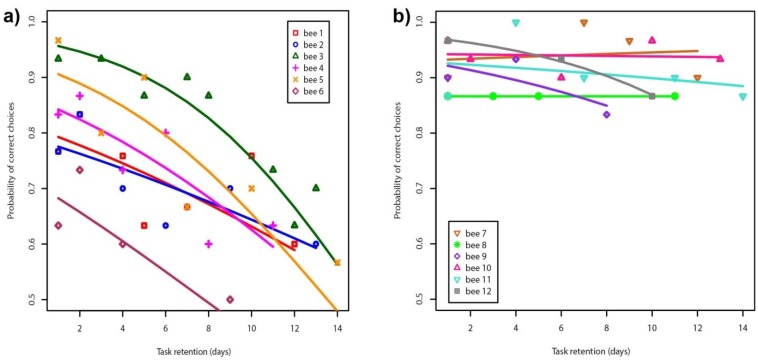
Probability of correct choices for a total of 12 bees performing a hard colour discrimination task (panel **a**, *n* = 6) and an easy discrimination task (panel **b**, *n* = 6) tested on different days during two weeks. Solid lines represent a logistic regression model fitted to the data corresponding to each individual bee.

Honeybees from Group 1 trained with differential conditioning to similar color stimuli were able to reliably learn the target color as mean accuracy on the initial day 1 test was 81.7% ± 12.2% s.d. (Figure 2a), and significantly different to chance expectation of 50% (one sample *t*-test, *t* = 6.333, d.f. = 5, *p* < 0.001). To analyze performance for choosing the correct target stimulus over several days we fitted individual logistic regression models to the percentage of correct choices from each bee performing either a hard or an easy colour discrimination task. This allowed for testing of changes in bee performance over multiple days using a Generalised Linear Regression Model (GLM) with a logit link function and assuming a response variable binomially distributed (logistic regression) fitted to the percentage of correct choices recorded for each individual bee. Regression analyses were done using the *glm* routine available in the statistical package R v 3.0.2 [32].

When these bees were tested on subsequent days, the data shows that whilst honeybees can perform a task that requires the discrimination between similar colors following differential conditioning, such a capacity to recall this task decays with increasing time (Figure 2a) as there was a significant difference in behavior considering binomially distributed logistic regression models Table 1. By comparison, bees in Group 2 were able to both reliably discriminate between stimuli on day one, and continue choosing the correct target color to which they were trained during tests for several days (Figure 2b), and there was no significant reduction in bee choices with increasing time (Table 1). The Group 2 result is consistent with previous reports that honeybees form a long term memory for easy color discrimination tasks [25], and suggests a potential difference in the reliability of honeybee memory for recalling a visual problem depending upon the perceptual difficulty of the task. This finding is thus consistent with the suggestion that factors like the complexity of a learning task or the salience of cues can influence the reliability with wish tasks can be recalled [27]. An alternative explanation to the interpretation that honeybee memory for similar colors may decay with time (Figure 2a), is that the required mechanism of motivating a free flying bee to participate in an experiment on each test day with brief absolute conditioning may interfere with the memory trace. We do not think this is a likely explanation of our results because in color learning experiments with differential conditioning it is possible to conduct non-rewarded testing multiple times with honeybees on the same day, with motivation rewards on the target between tests, and the capacity of the bees to choose the correct target stimulus is not significantly different over several different tests [33]. Furthermore, the biological context in which bees may require memory of similar colors is well mapped by our experiment design. An individual bee that on one day has learnt to discriminate between two similar colors because only one is rewarding and the alternative is a mimic [3,10,15,22,24], on subsequent days is likely to encounter some rewarding type flowers before having to remember the previously acquired fine discrimination task (especially in the case of mimics). In this situation, which does require memory of a learnt fine color task, free flying honeybees have a drop in performance with increasing time (Figure 2a) which is of high value to understand the interactions of pollinators and plants. However, it would be useful for future experiments to dissect the possibilities of memory decay and/or interference effects further considering time, and especially if there may be interaction between such factors on bee choices in complex environments.

**Table 1:** Parameters and 95% confidence intervals of the logistic regression models fitted to each one of the *n* = 12 bees used during the study. The model describes changes in the percentage of correct choices as a function of time for two different colour discrimination tasks, and statistically tests the significance of the slope. **^†^** Confidence intervals constructed based on a likelihood-based profile. **^§^** *p*-values calculated from differences in deviance values. ** Significant slopes at α = 0.05.

Bee	Intercept	Task Retention (Days) ^†^	*p*-Value ^§^
*Hard task*			
1 (7 days)	1.32 (0.75, 0.192)	−0.06 (−0.14, 0.015)	0.111
2 (6 days)	1.31 (0.74, 1.91)	−0.07 (−0.15, 0.00)	0.066
3 (9 days)	3.31 (2.44, 4.33)	−0.22 (−0.31, −0.14)	<0.000 **
4 (6 days)	1.80 (1.15, 2.53)	−0.13 (−0.23, −0.03)	0.01 **
5 (7 days)	2.44 (1.72, 3.26)	−0.18 (−0.26, −0.10)	<0.000 **
6 (5 days)	0.88 (0.27, 1.51)	−0.11 (−0.22, −0.00)	0.04 **
*Easy task*			
7 (4 days)	2.60 (1.31, 4.39)	0.02 (−0.17, 0.21)	0.802
8 (4 days)	1.87 (1.04, 2.82)	5.11 × 10^−17^ (0.14, 0.15)	1.000
9 (3 days)	2.57 (1.37, 4.1)	−0.10 (−0.35, 0.12)	0.371
10 (5 days)	2.80 (1.76, 4.15)	−0.01 (−0.16, 0.14)	0.916
11 (5 days)	2.56 (1.56, 3.80)	−0.04 (−0.16, 0.08)	0.532
12 (3 days)	3.60 (1.97, 6.10)	−0.17 (−0.45, 0.05)	0.142

One reason that might explain why bees may have different types of memory recall for easy and difficult color learning and discrimination tasks is that it appears these different types of visual problems may stimulate different pathways in the bee brain [28]. Specifically, whilst easy color tasks are learnt quickly and are processed by outer lobula, and both inner and outer medulla neurons that project to the posterior protocerebrum in the bee brain, for fine color tasks there appears to be a different color pathway activating neurons via the mushroom body and lateral protocerebrum pathway [28,34,35]. Thus different tasks appear to be solved in different ways by bees, and may thus have different dynamics for how memory is recalled. Further work on the regions of the bee brain that might underpin the behavioral evidence we observe would be of high value, although a current difficulty is measuring the apparent dynamic responses of free flying bees to color stimuli that occur over several hours during training, and the need to record from multiple regions of the bee brain during that learning process.

A number of studies have shown that for color tasks that have low chromatic contrast less than about 0.1 hexagon units that honeybees find the task perceptually very difficult. For example, individual honeybees will trade off speed for accuracy [36], or demonstrate variable individual behavior when required to reverse learn a task that initially took a long time to learn [29]. This current study reinforces that color stimuli are very useful for quantifying the perceptual difficulty of a visual task, which might provide important avenues for future research into the dynamics of honeybee memory in complex environments [27]. Interestingly a robust long term memory of color has also been reported in hawk moths, although the results vary depending upon whether color stimuli matched innate preferences (where color memory was very reliable) or did not match preferences (where color memory was less accurate) [37]. The effects of color similarity on hawk months also show that very similar colors are more likely to be confused in these insects as they switch to positional cues if available, whilst dissimilar colors are reliably chose based only on visual information [38]. The findings have important implications for understanding why flowers may have evolved salient color signals to avoid perceptual errors by bees or other pollinators [13,15]. Whilst it appears clear that bees learn color tasks very accurately with differential conditioning and aversion conditioning with quinine [15,22,39,40], such learning takes time to acquire, and retaining the information appears difficult for longer than a couple of days, so it is beneficial for flowering plants that offer rewards to promote flower constancy to present signals that are easily processed by the bees’ visual system. Exploiting this feature of bee task learning and recall on subsequent days may explain how some flowers that do not contain rewards [3] are able to obtain pollination services by bees by mimicking rewarding flowers.

## 4. Conclusions

Foraging insects like honeybees live and forage in complex natural conditions. Using perceptually similar or dissimilar colors in discrimination tasks, with independent groups of bees, it is shown that whilst easy visual tasks are recalled for many days after training, perceptually difficult tasks are often forgotten by free flying bees after several days. This suggests that bees readily forget hard problems, which potentially allows mimic flowers to gain pollination in complex natural environments. However, work to build a bridge between psychophysics experiments as reported here, and how insects respond to flowers with lower rewards in natural environments, would be of high value.
